# Application of Artificial Neural Network to Preoperative ^18^F-FDG PET/CT for Predicting Pathological Nodal Involvement in Non-small-cell Lung Cancer Patients

**DOI:** 10.3389/fmed.2021.664529

**Published:** 2021-04-22

**Authors:** Silvia Taralli, Valentina Scolozzi, Luca Boldrini, Jacopo Lenkowicz, Armando Pelliccioni, Margherita Lorusso, Ola Attieh, Sara Ricciardi, Francesco Carleo, Giuseppe Cardillo, Maria Lucia Calcagni

**Affiliations:** ^1^Unità Operativa Complessa (UOC) di Medicina Nucleare, Dipartimento di Diagnostica per Immagini, Radioterapia Oncologica ed Ematologia, Fondazione Policlinico Universitario A. Gemelli IRCCS, Rome, Italy; ^2^Unità Operativa Complessa (UOC) di Radioterapia Oncologica, Dipartimento di Diagnostica per Immagini, Radioterapia Oncologica ed Ematologia, Fondazione Policlinico Universitario A. Gemelli IRCCS, Rome, Italy; ^3^Department of Occupational and Environmental Medicine, Istituto Nazionale Assicurazione Infortuni sul Lavoro (INAIL), Rome, Italy; ^4^Nuclear Medicine Department, Jordanian Royal Medical Services, Amman, Jordan; ^5^Department of Cardiothoracic Surgery, S. Orsola-Malpighi University Hospital, Bologna, Italy; ^6^Unit of Thoracic Surgery, San Camillo Forlanini Hospital, Rome, Italy; ^7^Dipartimento Universitario di Scienze Radiologiche ed Ematologiche, Università Cattolica del Sacro Cuore, Rome, Italy

**Keywords:** PET/CT, 18F-FDG, non-small-cell lung cancer, artificial neural network, nodal staging

## Abstract

**Purpose:** To evaluate the performance of artificial neural networks (aNN) applied to preoperative ^18^F-FDG PET/CT for predicting nodal involvement in non-small-cell lung cancer (NSCLC) patients.

**Methods:** We retrospectively analyzed data from 540 clinically resectable NSCLC patients (333 M; 67.4 ± 9 years) undergone preoperative ^18^F-FDG PET/CT and pulmonary resection with hilo-mediastinal lymphadenectomy. A 3-layers NN model was applied (dataset randomly splitted into 2/3 training and 1/3 testing). Using histopathological reference standard, NN performance for nodal involvement (N0/N+ patient) was calculated by ROC analysis in terms of: area under the curve (AUC), accuracy (ACC), sensitivity (SE), specificity (SP), positive and negative predictive values (PPV, NPV). Diagnostic performance of PET visual analysis (N+ patient: at least one node with uptake ≥ mediastinal blood-pool) and of logistic regression (LR) was evaluated.

**Results:** Histology proved 108/540 (20%) nodal-metastatic patients. Among all collected data, relevant features selected as input parameters were: patients' age, tumor parameters (size, PET visual and semiquantitative features, histotype, grading), PET visual nodal result (patient-based, as N0/N+ and N0/N1/N2). Training and testing NN performance (AUC = 0.849, 0.769): ACC = 80 and 77%; SE = 72 and 58%; SP = 81 and 81%; PPV = 50 and 44%; NPV = 92 and 89%, respectively. Visual PET performance: ACC = 82%, SE = 32%, SP = 94%; PPV = 57%, NPV = 85%. Training and testing LR performance (AUC = 0.795, 0.763): ACC = 75 and 77%; SE = 68 and 55%; SP = 77 and 82%; PPV = 43 and 43%; NPV = 90 and 88%, respectively.

**Conclusions:** aNN application to preoperative ^18^F-FDG PET/CT provides overall good performance for predicting nodal involvement in NSCLC patients candidate to surgery, especially for ruling out nodal metastases, being NPV the best diagnostic result; a high NPV was also reached by PET qualitative assessment. Moreover, in such population with low *a priori* nodal involvement probability, aNN better identify the relatively few and unexpected nodal-metastatic patients than PET analysis, so supporting the additional aNN use in case of PET-negative images.

## Introduction

The evaluation of lymph nodal status is of paramount importance for selecting the optimal therapeutic approach in patients with non-small-cell lung cancer (NSCLC), with N0 and N1 patients addressed to surgery (when clinically feasible), and N3 ones to non-surgical approaches, while N2 patients still have more controversial therapeutic options (1, 2). 18-Fluorine-Fluorodeoxyglucose Positron Emission Tomography/Computed Tomography (^18^F-FDG PET/CT) is widely used for nodal staging in NSCLC patients, being recommended by the National Comprehensive Cancer Network (NCCN) guidelines (1). ^18^F-FDG PET/CT shows an overall good accuracy for nodal evaluation with sensitivity and specificity values ranging from 72 to 90% and from 81 to 95%, respectively (1–6). More recently, machine learning methods have been applied to ^18^F-FDG PET/CT as an advanced and innovative analysis tool in NSCLC patients for staging, treatment evaluation and prognostic stratification (7–10).

Neural Networks (NN) represent an application of machine learning based on an artificial reinterpretation of the human brain structure, that relies on the use of numerous layers of “neurons.” Each neuron is characterized by a specific weight and importance in the context of the whole network. Similarly, each layer receives data, calculates scores and passes the output of the analysis to the next layer in a self-learning process. This architecture has been recently widely used in the context of biomedical imaging research and radiation oncology, aiming to predict clinical outcomes and enrich diagnostic information, describing the interactions and complex simultaneous relationships of variables belonging to different domains (11–13). Growing, although still limited, literature evidence has explored the application of NN to ^18^F-FDG PET/CT for predicting nodal involvement in NSCLC patients, but burdened by differences in clinical and procedural aspects (14–17).

Aim of our study was to evaluate the performance of artificial neural network (aNN) applied to preoperative ^18^F-FDG PET/CT for predicting pathological nodal involvement in clinically resectable NSCLC patients.

## Materials and Methods

### Study Population

We retrospectively reviewed medical records of all consecutive patients referred to the PET/CT center of “Fondazione Policlinico Universitario Agostino Gemelli IRCCS” in Rome by a local Thoracic Surgery Unit between January 2007 and December 2017 for pulmonary lesions' evaluation. We included only patients with: (1) malignant pulmonary lesions histologically proven as NSCLC; (2) judged resectable at pre-operative Multidisciplinary Tumor Board evaluation (including those with single N2 station at pre-operative invasive mediastinal staging); (3) undergone lung resection and hilo-mediastinal lymphadenectomy; (4) not addressed to induction chemotherapy and/or radiotherapy. Exclusion criteria were: all patients not fitting the inclusion criteria; with proven N2 multistation or N3 at invasive mediastinal staging procedures. A set of clinical, anatomic, metabolic and histopathological data were retrospectively collected. Pathological TNM staging was defined according to the 8th staging system edition (18, 19). This retrospective study was approved by the local institution's ethics committee (Comitato Etico Lazio 1). For each patient, PET/CT imaging was performed in the clinical routine with written informed consent.

### ^18^F-FDG PET/CT Image Acquisition and Interpretation

All PET/CT were acquired according to standard protocol (6 h fasting-state, blood glucose levels <150 mg/dl; acquisition time of 60 ± 10 min post-injection of 185–370 MBq of ^18^F-FDG, according to BMI), using an integrated 3D PET/CT device (Gemini GXL by Philips Medical System, Cleveland, Ohio or Biograph mCT by Siemens Healthineers, Chicago, Illinois) with a low-dose unenhanced CT scan (120 kV, 50–80 mA) for anatomical localization and attenuation correction. All PET images (reconstructed with iterative algorithms) were evaluated by two independent nuclear medicine physicians (ST, VS), blinded to the final pathological TNM staging, using a dedicated fusion and display software (Syngo.via MM Oncology software; Siemens Medical Solutions). For primary lung tumor evaluation, a qualitative analysis was performed: PET was scored as positive if ^18^F-FDG uptake was equal or higher than the mediastinal blood-pool, as negative if lower. A semiquantitative analysis was also performed: for lesions segmentation, a fixed relative threshold method was adopted and a 3D volumetric region of interest (VOI) was drawn semi-automatically over the primary tumor on fused PET/CT images, with a fixed threshold of 40% of the maximum standardized uptake value. Then, the following tumor semiquantitative parameters were extracted, applying the EQ·PET quantification technology (20): maximum and mean standardized uptake values (SUVmax, SUVmean), metabolic tumor volume (MTV, expressed in cm^3^) and total lesion glycolysis (TLG, defined as the product of SUVmean and MTV). The anatomical consistency of tumor delineation was visually checked and volumetric region of interest was adjusted manually, if needed. For nodal evaluation, a visual patient-based PET nodal status was defined: any lymph node with ^18^F-FDG uptake ≥ mediastinal blood-pool was classified as PET positive; each patient with at least one positive lymph node was scored as PET positive (PET N+), otherwise as negative (PET N0). Moreover, for each patient a PET nodal staging (PET N0, N1, N2, or N3) was defined, according to sites of PET positive nodes and the 8th edition of TNM staging. Any disagreement was resolved by consensus. Histological nodal status was used as reference standard to verify PET results.

### Neural Network Development

The collected clinical, anatomic, metabolic, and histopathological features were used as input parameters of the model: patients' age and gender, tumor size and location (as right/left lung, upper/lower lobes, and central/peripheral), PET tumor visual result and semiquantitative parameters, PET nodal status, PET nodal staging, tumor histotype and grading. Categorical features were binarized and numerical features were *Z*-standardized. The dataset was randomly split into 2/3 training and 1/3 testing, being the sample size numerous enough to perform hold-out validation instead of cross-validation. Relevant features to outcome of interest (i.e., surgically-proven nodal status) were selected with Boruta algorithm on the training set (21). A NN based model was then realized with the selected features. Considering the sample size, the events distribution and the number of selected features, a 3-layers neural network (12, 6, and 2 activation neurons, respectively) was trained on the training set with the Boruta-selected features as input. Network training specifications were as follows: the first two layers had activation function ReLu, while the third layer (classification layer) had Softmax. Categorical cross-entropy was the loss function and Adam was the optimizer. The model was trained for 500 epochs with 150 batch size and 0.1 validation split. Classification performance of the trained network in predicting nodal involvement was evaluated on the testing set applying the Receiver Operating Characteristic (ROC) analysis, using histological nodal status as reference standard. Logistic regression (LR) model was also trained on the training set after Akaike information criterion (AIC)-based stepwise selection on the Boruta-selected features.

### Statistical Analysis

Continuous variables were expressed as mean (with standard deviation) or median (with range) and categorical data as a percentage. Comparison between training and testing groups in collected features were performed using Mann–Whitney/Chi-square test for continuous and categorical data, respectively. On ROC analysis, the NN diagnostic performance for nodal involvement (on both training and testing sets) was calculated in terms of Area Under the Curve (AUC) and classification matrix at the Youden-index classification threshold were computed: accuracy (ACC), sensitivity (SE), specificity (SP), positive and negative predictive values (PPV and NPV). Diagnostic performances for nodal involvement (N0/N+) of the visual PET analysis and LR model (on both training and testing sets) were also assessed. PPV and NPV were calculated assuming that the individual pre-test probability of nodal metastatic disease was equal to the prevalence of pathological nodal involvement (pN+) found in our population. Results were reported with 95% Confidence Intervals (CIs). Statistical significance was set at *p* < 0.05. Statistical analyses were performed in R version 3.4 and Python version 3.7.

## Results

### Study Population

Finally, 540 consecutive clinically resectable NSCLC patients (333 males; mean age: 67.4 ± 9 years), who underwent pre-operative ^18^F-FDG PET/CT (44 ± 28 days before surgery) were selected. Table 1 reports the main characteristics of the study population. Among the 540 patients, 528 underwent lobectomy, nine bi-lobectomy and three atypical pulmonary resection. A total of 1,620 nodal stations (from station 2 to 11) were histologically evaluated (3 ± 1 stations per patient), with a total of 4,158 examined nodes (8 ± 5 nodes per patient); peribronchial nodes found in the resected lobe were also pathologically assessed in 439 patients. Histopathological nodal involvement was found in 108/540 (20%) patients: 45/108 staged as pN1 and 63/108 as pN2. Overall, in the total 540 patients, 80% resulted as pN0, 8.3% as pN1 and 11.7% as pN2. According to pathologic staging, 383 patients were classified as stage I (28 IA1; 152 IA2; 78 IA3; 125 IB), 74 stage II (13 IIA; 61 IIB), 80 stage III (70 IIIA; 10 IIIB), and 3 stage IVA (for pleural localizations).

**Table 1:** Main clinical, anatomic, metabolic, and histopathological characteristics of the study population (*n* = 540).

**Characteristics**	***N***
**Gender**	
Male	333 (61.7%)
Female	207 (38.3%)
**Age (years)**	
Mean ± SD	67.4 ± 9
**Tumor size (mm)**	
Mean ± SD	25.3 ± 14.3
**Tumor location**	
Right lung	293 (54.2%)
Upper lobes^a^	348 (64.4%)
Central^b^	146 (27%)
**Tumor visual PET result**	
Positive	452 (83.7%)
Negative	88 (16.3%)
**Tumor SUVmax**	
Mean ± SD	6.6 ± 5.6
**Tumor SUVmean**	
Mean ± SD	4.2 ± 3.4
**MTV (cm^3^)**	
Mean ± SD	7.6 ± 16.7
**TLG**	
Mean ± SD	52.6 ± 182.1
**Nodal visual PET result**	
Nodal status	479 N0 (88.7%), 61 N+ (11.3%)
Nodal staging	479 N0 (88.7%), 27 N1 (5%), 34 N2 (6.3%)
**Histology**	
Adenocarcinoma	385 (71.3%)
Squamous cell carcinoma	89 (16.5%)
Others	66 (12.2%)
**Grading**	
G1	73 (13.5%)
G1–G2, G2	26 (4.8%), 201 (37.2%)
G2–G3, G3	68 (12.6%), 167 (31%)
G4	5 (0.9%)
**Pathological N result (pN)**	
N0	432 (80%)
N1	45 (8.3%)
N2	63 (11.7%)

*SD, standard deviation; MTV, metabolic tumor volume; TLG, total lesion glycolysis*.

a *The right middle lobe and lingula were included in the upper lobes location*.

b *The lung lesion was defined as central if located in the inner one-third of the lung parenchyma, and as peripheral if located in the outer two-thirds of lung parenchyma*.

### Neural Network Analysis

**Figure 1:**
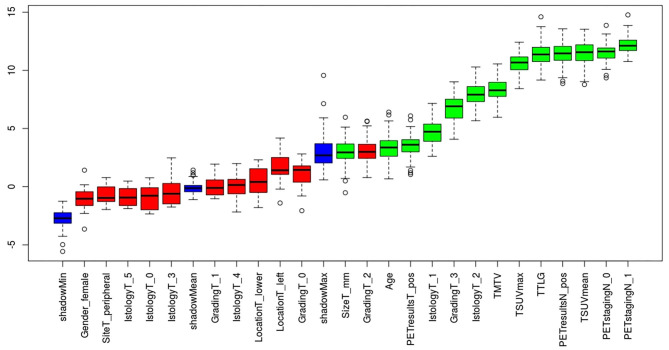
Relevant features (*highlighted in green*) to the outcome of interest (pathological nodal involvement) selected with Boruta algorithm.

**Figure 2:**
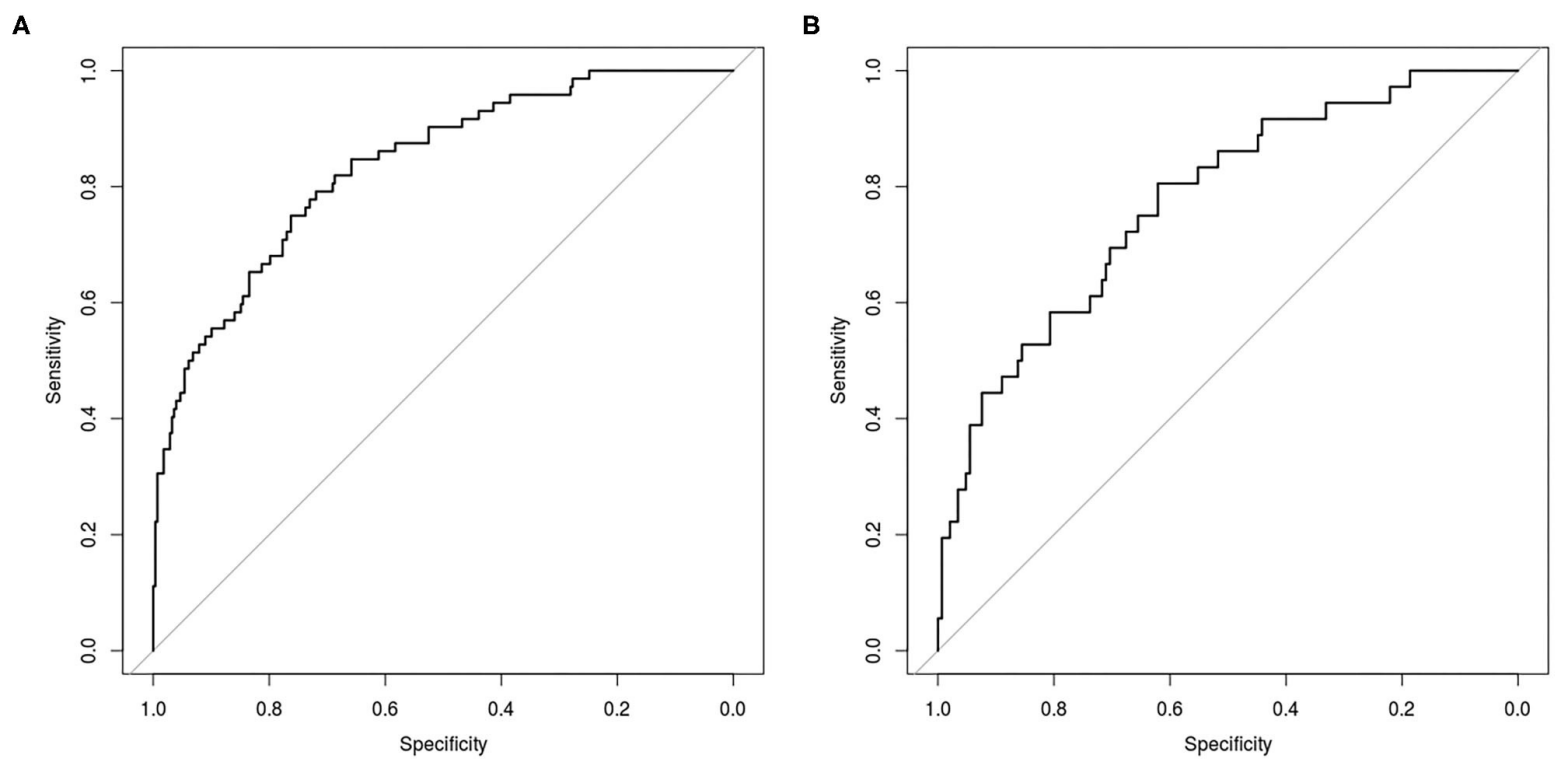
Receiver Operating Characteristic (ROC) curve for prediction of nodal involvement according to NN analysis in the training set **(A)** and in the testing set **(B)**.

No features differences (*p* > 0.05) were observed between training and testing sets (Table 2), that also showed the same proportion of pN0 and pN+ patients. From the set of collected features, Boruta algorithm selected 13 relevant as input parameters (Figure 1): patients' age, tumor size, PET tumor parameters (visual result, SUVmax, SUVmean, TLG, MTV), patient-based PET nodal status (as N0/N+) and PET nodal staging (as N0/N1/N2), tumor histotype (adenocarcinoma, squamous cell carcinoma) and grading (G3). The NN was then trained with all Boruta-selected features as input variables. From ROC analysis, NN diagnostic performance for nodal involvement (N+/N0) for the training and testing sets were: AUC = 0.849 (95%CI: 0.751–0.838), ACC = 0.80 (95%CI: 0.75–0.84), SE = 0.72 (95%CI: 0.60–0.82), SP = 0.81 (95%CI: 0.76–0.86), PPV = 0.50 (95%CI: 0.40–0.60), NPV = 0.92 (95%CI: 0.88–0.95), and AUC = 0.769 (95%CI: 0.699–0.827), ACC = 0.77 (95%CI: 0.70–0.83), SE = 0.58 (95%CI: 0.41–0.74), SP = 0.81 (95%CI: 0.74–0.87); PPV = 0.44 (95%CI: 0.30–0.59), NPV = 0.89 (95%CI: 0.82–0.93), respectively (Table 3 and Figure 2).

**Table 2:** Comparison in collected features between training and testing groups.

**Feature**	***p*-value**
Age^a^	0.17
Gender (*male/female*)	0.31
Location T (*right/left lung*)	0.22
Location T (*upper/lower lobe*)	0.57
Site T (*central/peripheral*)	1.00
Histology T0 (*neuroendocrine tumor*)^b^	0.95
Histology T1 (*adenocarcinoma*)^b^	0.94
Histology T2 (*squamous cell carcinoma*)^b^	0.97
Histology T3 (*adeno-squamous carcinoma*)^b^	1.00
Histology T4 (*pleomorphic carcinoma*)^b^	0.84
Histology T5 (*poorly differentiated carcinoma*)^b^	0.35
Grading T0 (*G1–G2*)^b^	0.65
Grading T1 (*G1*)^b^	0.27
Grading T2 (*G2*)^b^	0.61
Grading T3 (*G3*)^b^	0.25
Size T (*mm*)^a^	0.52
PET result T (*negative/positive*)	0.25
T SUVmax^a^	0.35
T SUVmean^a^	0.25
T TLG^a^	0.45
T MTV^a^	0.83
PET result N (*negative/positive*)	0.51
PET staging N0 (*N0/not N0*)^b^	0.51
PET staging N1 (*N1/not N1*)^b^	1.00

*T, tumor; N, nodal*.

a *Numerical features were Z-standardized*.

b *Categorical features were binarized*.

**Table 3:** Diagnostic performance of neural network, logistic regression, and visual ^18^F-FDG PET/CT analysis for pathological nodal involvement.

	**Training set (*****n*** **= 356)**		**Test set (*****n*** **= 184)**		**All dataset (*n* = 540)**
	**NN**	**LR**	**NN**	**LR**	**PET visual analysis**
AUC (95%CI)	0.849 (0.751–0.838)	0.795 (0.700–0.800)	0.769 (0.699–0.827)	0.763 (0.669–0.820)	n.a.
ACC (95%CI)	0.80 (0.75–0.84)	0.75 (0.70–0.80)	0.77 (0.70–0.83)	0.77 (0.70–0.83)	0.82 (0.78–0.85)
SE (95%CI)	0.72 (0.60–0.82)	0.68 (0.56–0.73)	0.58 (0.41–0.74)	0.55 (0.38–0.72)	0.32 (0.24–0.42)
SP (95%CI)	0.81 (0.76–0.86)	0.77 (0.72–0.82)	0.81 (0.74–0.87)	0.82 (0.75–0.88)	0.94 (0.91–0.96)
PPV (95%CI)	0.50 (0.40–0.60)	0.43 (0.34–0.53)	0.44 (0.30–0.59)	0.43 (0.29–0.59)	0.57 (0.45–0.69)
NPV (95%CI)	0.92 (0.88–0.95)	0.90 (0.86–0.94)	0.89 (0.82–0.93)	0.88 (0.81–0.93)	0.85 (0.81–0.88)

*NN, neural network; LR, logistic regression; AUC, area under the curve; CI, confidence interval; ACC, accuracy; SE, sensitivity; SP, specificity; PPV, positive predictive value; NPV, negative predictive value*.

### ^18^F-FDG PET/CT

**Figure 3:**
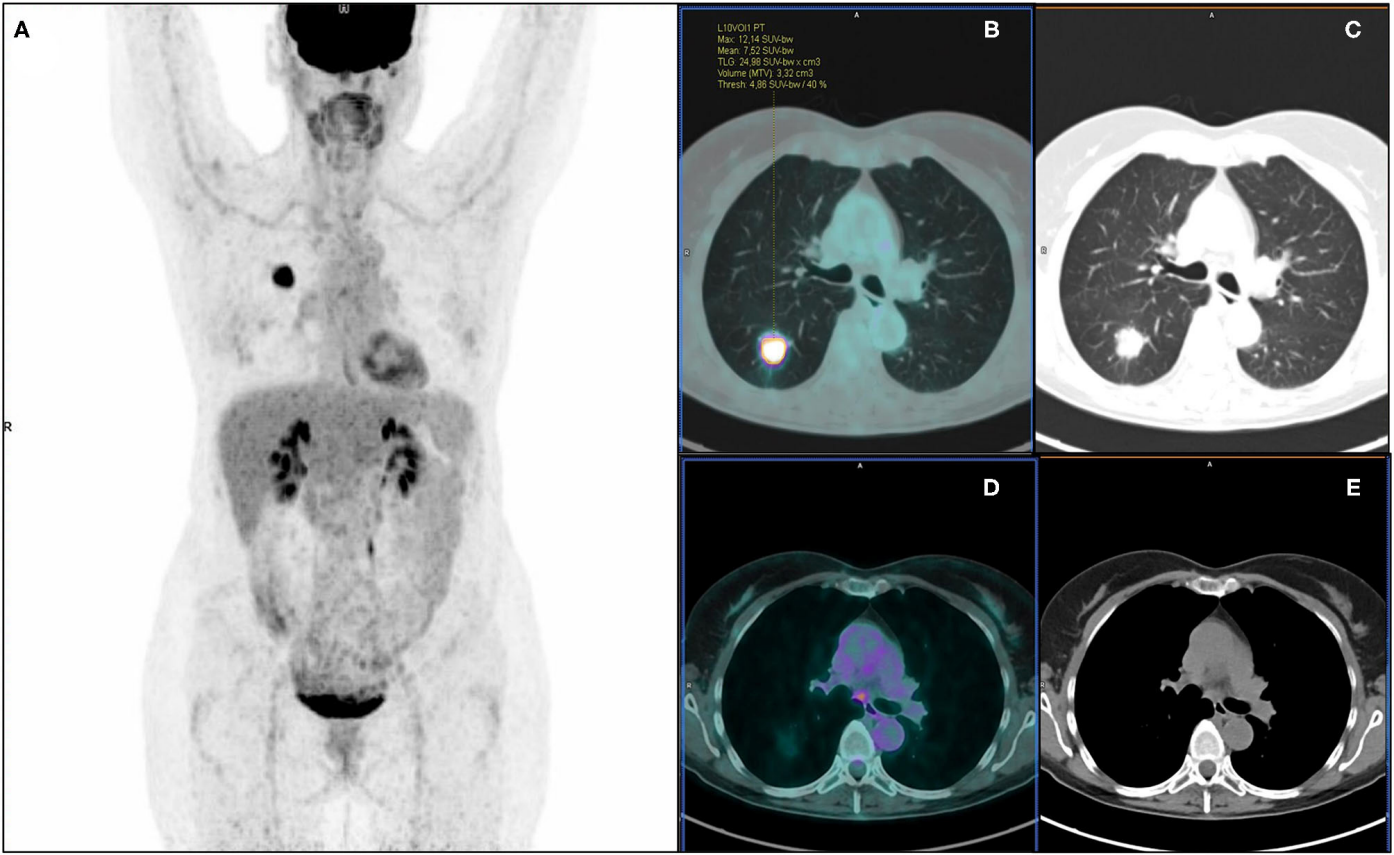
^18^F-FDG PET/CT maximum intensity projection **(A)**, transaxial fused **(B)**, and coregistered CT images **(C)** of a 57-year-old female with lung adenocarcinoma of the right lower lobe (maximum axial diameter: 30 mm), showing increased metabolic activity in the primary tumor lesion (SUVmax: 12.14; SUVmean: 7.52; MTV: 3.32 cm^3^; TLG: 24.98) and a focus of increased tracer uptake in a subcarinal mediastinal lymph node **(D,E)**. According to visual analysis, the patient was classified as PET positive for nodal involvement (PET N+). Further histopathological examination revealed no pathological nodal involvement (pN0, PET false-positive). Artificial NN correctly classified the patient as N0.

**Figure 4:**
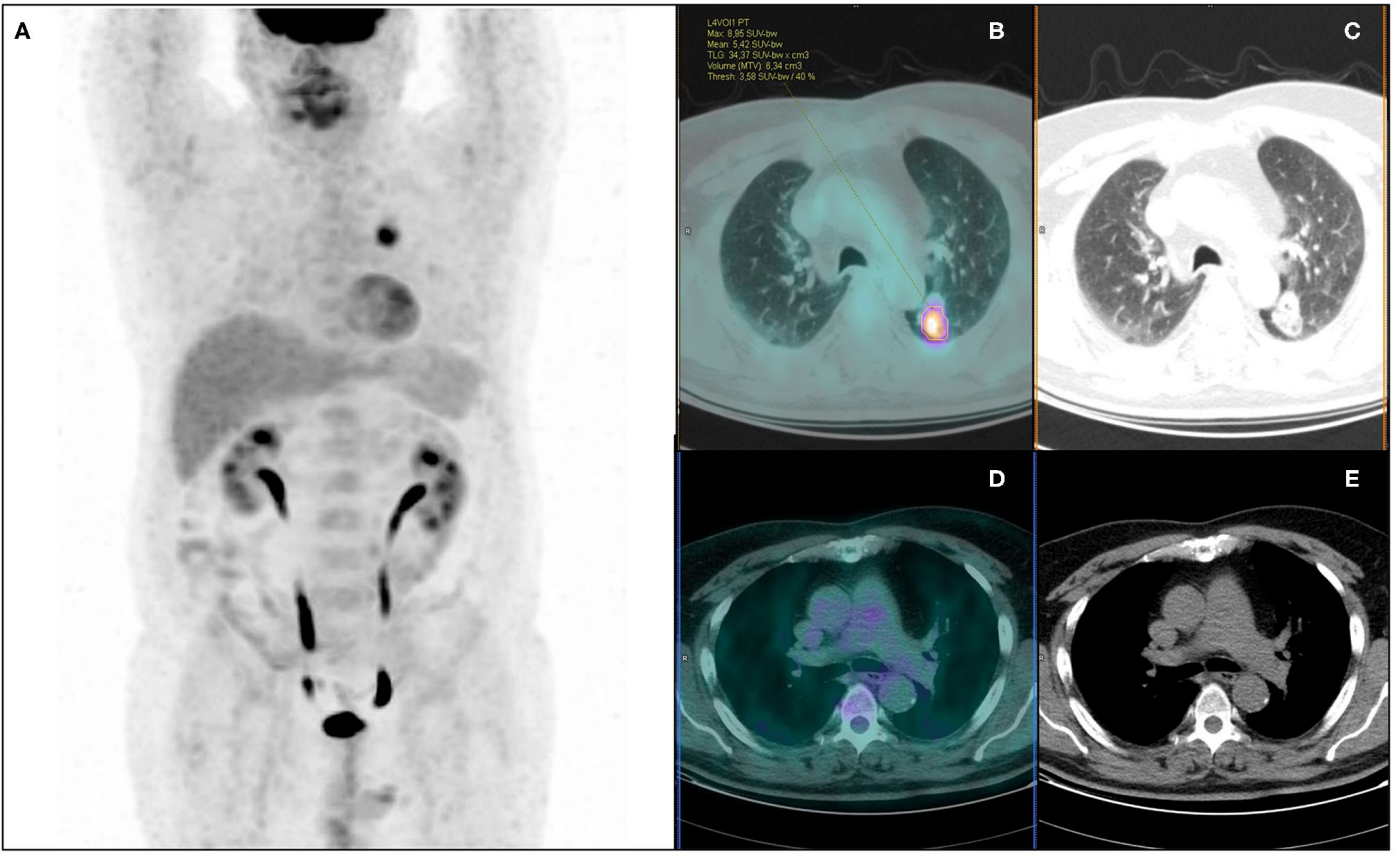
^18^F-FDG PET/CT maximum intensity projection **(A)**, transaxial fused **(B)**, and coregistered CT images **(C)** of a 64-year-old male with lung adenocarcinoma of the left lower lobe (maximum axial diameter: 32 mm), showing increased metabolic activity in the primary tumor lesion **(A)** (SUVmax: 8.95; SUVmean: 5.42; MTV: 6.34 cm^3^; TLG: 34.37), with no abnormal focus of increased tracer uptake in hilo-mediastinal lymph nodes **(D,E)**. According to visual analysis, the patient was classified as PET negative for nodal involvement (PET N0). Further histopathological examination revealed metastatic homolateral hilar nodes (pN+, PET false-negative). Artificial NN correctly classified the patient as N+.

On PET visual analysis, 479/540 patients were classified as N0: 406/479 with no pathological nodal involvement (pN0, PET true-negatives), 73/479 with at least one metastatic node (pN+, PET false-negatives). The remaining 61/540 patients were classified as PET positive for nodal involvement: 35/61 histologically confirmed (pN+, PET true-positives), 26/61 with no pathological nodes (pN0, PET false-positives). Diagnostic performance of PET visual analysis for nodal involvement (N0/N+) was: ACC = 0.82 (95%CI: 0.78–0.85), SE = 0.32 (95%CI: 0.24–0.42), SP = 0.94 (95%CI: 0.91–0.96), PPV = 0.57 (95%CI: 0.45–0.69), NPV = 0.85 (95%CI: 0.81–0.88) (Table 3). When considering PET nodal staging, among the 479 PET negative patients, 406/479 (84.8%) were correctly staged resulting pN0, 73/479 (15.2%) were upstaged resulting pN1 (30/73) or pN2 (43/73). Regarding the 61 PET positive patients, 27 were classified as PET N1 and 34 as PET N2. Among PET N1 patients, 11/27 were correctly staged resulting pN1, 8/27 were downstaged resulting pN0 and 8/27 were upstaged resulting pN2. Among PET N2 patients, 12/34 were correctly staged resulting pN2, 22/34 were downstaged resulting pN1 (4/22) or pN0 (18/22). PET/CT images of illustrative cases are reported in Figures 3, 4.

The LR model with stepwise selection based on AIC criteria gave the model in Table 4. Logistic regression diagnostic performance for nodal involvement at training and testing group were: AUC = 0.795 (95%CI: 0.700–0.800), ACC = 0.75 (95%CI: 0.70–0.80), SE = 0.68 (95%CI: 0.56–0.73), SP = 0.77 (95%CI: 0.72–0.82), PPV = 0.43 (95%CI: 0.34–0.53), NPV = 0.90 (95%CI: 0.86–0.94), and AUC = 0.763 (95%CI: 0.669–0.820), ACC = 0.77 (95%CI: 0.70–0.83), SE = 0.55 (95%CI: 0.39–0.72), SP = 0.82 (95%CI: 0.75–0.88), PPV = 0.43 (95%CI: 0.29–0.59), NPV = 0.88 (95%CI: 0.81–0.93), respectively (Table 3).

**Table 4:** Logistic regression model with stepwise selection based on AIC criteria.

**Variable**	**Coefficient**	**Standard error**	***p*-value**
Intercept	−2.071	1.480	0.1
Tumor histology (*squamous cell carcinoma*)	−1.595	0.518	0.002
Tumor grading (*G3*)	1.121	0.309	0.0003
PET tumor result (*positive*)	2.318	1.032	0.02
PET nodal result (*positive*)	1.436	0.473	0.002
PET nodal staging (*N1*)	1.038	0.724	0.1
Patient age	−0.029	0.017	0.08

## Discussion

Aim of our study was to evaluate the diagnostic performance of aNN to preoperative ^18^F-FDG PET/CT for predicting pathological nodal involvement in clinically resectable NSCLC patients. The main strength points of this study are: the largest lung cancer population on which NN were applied for the same aim; the use of the widest combination of clinical, anatomic, metabolic, and histopathological features as input parameters; the surgical lymphadenectomy as golden reference in all patients.

From our results, aNN provided overall good performance for predicting pathological nodal involvement with a diagnostic accuracy >75% at both training and testing sets; similar diagnostic performance on both datasets suggests that overfitting was successfully reduced, supporting the reliability of the results. NN showed higher specificity and NPV than sensitivity and PPV, providing the best diagnostic performance for ruling out nodal metastases. In this context, it has to be considered that the pre-test probability of nodal involvement (and in turn the positive and negative predictive values) mainly depends on the NSCLC clinical settings. Indeed, our population has low *a priori* probability of nodal involvement since deemed clinically resectable, as confirmed by the low prevalence of nodal-metastatic patients and the high NPV. On the other hand, preoperatively identifying the relatively few and unexpected nodal-metastatic patients assumes great relevance, since other treatment strategies rather than the planned up-front surgery can be considered. However, PET visual analysis provided a poor sensitivity, with occult lymph nodal metastases mainly due to small size of metastatic lymph nodes, nodal micro-metastases (22) or metastatic hilar nodes masked by the intense activity of close primary tumor (14, 23). Although suboptimal, the sensitivity provided by aNN resulted relevantly higher than visual analysis (72 vs. 32%, respectively), suggesting that aNN may reduce the chance of ^18^F-FDG PET/CT false negative results. From a practical point of view, this finding may support the additional use of aNN to the PET/CT reporting activity in case of visually negative images. This diagnostic advantage of aNN may be attributed to the intrinsic properties of this machine learning method, able to explore and recognize complex and generally non-linear relationships among multiple variables, obviously going beyond the PET visual assessment alone.

Analyzing the relevant features selected by Boruta algorithm as input parameters, the metabolic features were the most numerous (8/13) and the first ones in order of importance: PET nodal staging (N0/N1/N2) in the first position, followed by PET nodal status (N0/N+) and semiquantitative parameters. We may suppose that PET nodal staging resulted as the most relevant feature since it appears intrinsically more linked to the target output (i.e., pathological nodal status) than other variables, providing direct and complete information on nodal assessment (even more detailed than dichotomous PET nodal status). When considering the PET semiquantitative tumor-related parameters, their relevance seems to be expected, being widely reported in literature as predictive factors of pathological nodal involvement in NSCLC (24–31).

Visual analysis of primary lesion resulted the last relevant metabolic feature. This finding may be reasonably attributed to its dichotomous nature (uptake ≥ or < mediastinal blood-pool activity) compared to the continuous nature of semiquantitative parameters (wide range of uptake levels), so providing less detailed information on tumor metabolism. Among the anatomic variables, tumor size resulted the only relevant feature: it was already reported to be a predictive factor for nodal involvement (26, 32) since reflecting the T-classificator in the TNM staging, and the risk of lymph node involvement increases with the increase of T stage. Lastly, among histological variables, the relevance of G3 (grading-feature) appears in line with the expectations: high grading, reflecting high tumor aggressiveness, increases the risk of metastatic nodal involvement (33), as also observed in our study, with a higher rate of nodal-metastatic patients in G3 group than in well or moderately differentiated groups (34.1 vs. 11.3%). Finally, regarding tumor histotype, more nodal metastases in adenocarcinomas than in squamous cell carcinomas were observed in our population (21.8 vs. 10.1%), supposing that adenocarcinoma type would be more informative for aNN regarding the risk of nodal involvement. Nevertheless, adenocarcinoma resulted a relatively weaker input parameter than the other one, in line with literature, as no concordant and definitive results on the greater or lesser predictive role of one or the other histotype have emerged (26, 27, 34, 35).

In our study, the LR results were only slightly lower compared to NN. A neural network is more complex than LR since one can think of it as a subset of a neural network classifier. LR model can always be simulated using a NN with one hidden node with the identity activation function and one output node with zero bias and logistic sigmoid activation. This finding can suggest that, when applying aNN to ^18^F-FDG PET for predicting nodal metastases, the added value of modeling non-linear interactions is not sufficient to substantially increase the diagnostic performance, also given the strong association of input variables (primarily PET-related) with the outcome.

Few studies in literature evaluated the application of aNN to ^18^F-FDG PET/CT in NSCLC for predicting nodal involvement, with several differences in clinical and procedural aspects among single studies and when compared to our work. In particular, Vesselle et al. (14) and Toney et al. (15), investigating 133 NSCLC patients, reported a NN accuracy higher than accuracy of PET expert reader (87.3 and 99.2% vs. 73.5 and 72.4%, respectively). This result seems to outperform the performance reported in our study. However, both authors reported an increased PET accuracy and closer to NN performance (up to 92.2%) when N status was dichotomized in N0+N1 vs. N2+N3 disease. Moreover, it has to be considered that both studies are not comparable to our work due to several aspects (smaller population, inclusion of non-surgically treated patients, heterogeneous reference standard, higher rate of nodal-metastatic patients, fewer input parameters and without PET-volumetric ones) and, mainly, to the criteria used for PET visual nodal analysis, that likely affect the comparison between PET and NN performance. Indeed, nodes have been interpreted as benign or malignant according on the expert reader's clinical experience, taking into account also size, location, and activity of primary tumor and size of the most metabolically active node. On the contrary, we used a strictly metabolic, more standardized, reproducible and objective criterion, interpreting nodes as benign or malignant only based on the mediastinal blood-pool activity. Anyway, the value of adding morphological nodal information for PET interpretation appears negligible in our population of clinically resectable patients, being almost all nodes with short axis ≤ 10 mm. Among other two studies focused on the same topic (16, 17), only one compared NN with PET visual performance, reporting similar accuracy, higher NN sensitivity and lower NN specificity for predicting nodal involvement. However, differences in population and/or methodological aspects make both studies not directly comparable to our paper. Finally, our study used the widest combination of clinical, anatomic, metabolic and histopathological data as input parameters, while only morpho-anatomic and/or metabolic features were considered in all the four previous studies; in addition, none of these studies reported LR model for comparison with aNN performance.

We acknowledge some limitations of our study, mainly represented by its retrospective nature. Moreover, a selection bias has to be considered, since only NSCLC patients candidate to surgical resection were included in our analysis, inherently lowering the prevalence of lymph nodal involvement. This aspect could have made our results generalizable only to similar cohorts of NSCLC patients, also affecting the diagnostic performance, especially in terms of PPV (due to the low rate of true positive patients). On the other hand, selecting only patients with surgical nodal evaluation allowed a reliable and robust verification of our results. Finally, external data validation was not applied.

In conclusion, the application of aNN to preoperative ^18^F-FDG PET/CT, along with clinical, anatomic and histopathological features, provides overall good performance for predicting pathological nodal involvement in clinically resectable NSCLC patients, especially for ruling out nodal metastases. Compared to visual PET analysis, aNN seem able to reduce the chance of PET false negative results; this finding assumes particular relevance in a population of clinically resectable NSCLC patients, with low *a priori* probability of nodal involvement, when the identification of the relatively few and unexpected nodal-metastatic patients may change their planned treatment strategies and also impact on prognosis. From a practical point of view, our observations may support the additional use of aNN to the PET/CT reporting activity in case of visually negative images. The application of aNN for categorizing pathological nodal involvement in N1 vs. N2 disease is topic of further ongoing analyses.

## Data Availability Statement

The raw data supporting the conclusions of this article will be made available by the authors, without undue reservation.

## Ethics Statement

The studies involving human participants were reviewed and approved by Comitato Etico Lazio 1. The ethics committee waived the requirement of written informed consent for participation.

## Author Contributions

MC, GC, ST, and AP were involved in conception and design of the study. ST, VS, ML, SR, and FC were involved in acquisition of PET/CT and clinical data. ST, VS, and OA analyzed PET/CT data. LB and JL performed logistic regression and neural network analyses. ST and VS conducted a literature research. ST, LB, and JL drafted the manuscript. MC, GC, and AP critically revised the manuscript for important intellectual content. All authors revised the final manuscript and gave their final approval for manuscript submission.

## Conflict of Interest

The authors declare that the research was conducted in the absence of any commercial or financial relationships that could be construed as a potential conflict of interest.
